# Casemix system in Nordic countries

**DOI:** 10.1186/1472-6963-12-S1-I2

**Published:** 2012-11-21

**Authors:** Olafr Steinum

**Affiliations:** 1 Rörviksvägen 19, SE-451 77 Uddevalla, Sweden

## 

The five Nordic countries are characterized by stable political systems based on consensus and interactive contracts between the populations and their governments. There has been a prosperous development through more than half a century, which also has resulted in a model of ’good health care for all’. However from late part of 1980s and during the 1990s it was realised that the cost of health care escalated beyond what was considered to be sustainable.

The concept of Casemix was introduced to all the Nordic countries over a few years in the 1990s, but the implementation of Casemix systems differed considerably between the five countries.

From 1966 the NOMESCO (Nordic Medico-Statistical Committee) presented statistical analyses to facilitate comparisons between the Nordic countries, and based on experiences from the NOMESCO collaboration, the Nordic Centre for Classifications in Health Care was established as a WHO Collaborating Centre in 1987.

The Centre has been a common arena to develop and manage health classifications (NCSP, NCECI) and the NordDRG Casemix grouper, as well as support to the WHO ICD-10 update process with views from the Nordic medical profession.

At the presentation elaboration on the historical development will be given with focus on the various paths of development, which the Casemix concept took in each of the Nordic countries. The differences in governance (state engagement or not) are discussed, as is the need for knowledge and education. Innovation and future trends will be mentioned.

